# Intestinal permeability is associated with aggravated inflammation and myofibroblast accumulation in Graves’ orbitopathy: the MicroGO study

**DOI:** 10.3389/fendo.2023.1173481

**Published:** 2023-11-30

**Authors:** Aline C. Fenneman, Anne H. van der Spek, Annick Hartstra, Stefan Havik, Anne Salonen, Willem M. de Vos, Maarten R. Soeters, Peeroz Saeed, Max Nieuwdorp, Elena Rampanelli

**Affiliations:** ^1^ Department of (Experimental) Vascular Medicine, Amsterdam Cardiovascular Sciences (ACS), Amsterdam University Medical Centre (UMC), University of Amsterdam, Amsterdam, Netherlands; ^2^ Department of Endocrinology and Metabolism, Amsterdam Gastroenterology Endocrinology & Metabolism (AGEM), Amsterdam University Medical Centre (UMC), University of Amsterdam, Amsterdam, Netherlands; ^3^ Human Microbiome Research Program, Faculty of Medicine, University of Helsinki, Helsinki, Finland; ^4^ Laboratory of Microbiology, Wageningen University & Research, Wageningen, Netherlands; ^5^ Department of Ophthalmology, Amsterdam University Medical Centre (UMC), Amsterdam, Netherlands

**Keywords:** gut microbiota, spatial transcriptomics, 16S rRNA, autoimmune disease, endocrinology

## Abstract

**Background:**

Graves’ disease (GD) and Graves’ orbitopathy (GO) result from ongoing stimulation of the TSH receptor due to autoantibodies acting as persistent agonists. Orbital pre-adipocytes and fibroblasts also express the TSH receptor, resulting in expanded retro-orbital tissue and causing exophthalmos and limited eye movement. Recent studies have shown that GD/GO patients have a disturbed gut microbiome composition, which has been associated with increased intestinal permeability. This study hypothesizes that enhanced intestinal permeability may aggravate orbital inflammation and, thus, increase myofibroblast differentiation and the degree of fibrosis.

**Methods:**

Two distinct cohorts of GO patients were studied, one of which was a unique cohort consisting of blood, fecal, and retro-orbital tissue samples. Intestinal permeability was assessed by measuring serum lipopolysaccharide-binding protein (LBP), zonulin, TLR5, and TLR9 ligands. The influx of macrophages and accumulation of T-cells and myofibroblast were quantified in orbital connective tissue. The NanoString immune-oncology RNA targets panel was used to determine the transcriptional profile of active fibrotic areas within orbital sections.

**Results:**

GO patients displayed significantly higher LBP serum concentrations than healthy controls. Within the MicroGO cohort, patients with high serum LBP levels also showed higher levels of zonulin and TLR5 and TLR9 ligands in their circulation. The increased intestinal permeability was accompanied by augmented expression of genes marking immune cell infiltration and encoding key proteins for immune cell adhesion, antigen presentation, and cytokine signaling in the orbital tissue. Macrophage influx was positively linked to the extent of T cell influx and fibroblast activation within GO-affected orbital tissues. Moreover, serum LBP levels significantly correlated with the abundance of specific Gram-negative gut bacteria, linking the gut to local orbital inflammation.

**Conclusion:**

These results indicate that GO patients have enhanced intestinal permeability. The subsequent translocation of bacterial compounds to the systemic circulation may aggravate inflammatory processes within the orbital tissue and, as a consequence, augment the proportion of activated myofibroblasts, which actively secrete extracellular matrix leading to retro-orbital tissue expansion. These findings warrant further exploration to assess the correlation between specific inflammatory pathways in the orbital tissue and the gut microbiota composition and may pave the way for new microbiota-targeting therapies.

## Introduction

Graves’ disease (GD), characterized by TSH-receptor stimulating antibodies and increased thyroid hormone serum levels, is an autoimmune disease affecting roughly 3% of the general population (1). GD is the most common form of hyperthyroidism (1) and up to 40% [CI 0.32 – 0.48] of GD patients have clinically apparent abnormalities of orbit soft tissue, known as Graves’ orbitopathy (GO) or thyroid eye disease (TED) (2).

Manifestations of GD/GO result from a B cell-mediated autoimmune response against the thyrotropin receptor (TSHR), resulting in the plasma cell production of autoantibodies targeting the thyrotropin receptor (TRAb). These autoantibodies bind and stimulate the TSH receptor resulting in excess secretion of thyroid hormones, namely tri-iodothyronine (T3) and thyroxine (T4), thus causing the clinical manifestations (3, 4). TSHR is also expressed in extra-thyroidal tissue, including the orbital fat tissue and extra-ocular muscles; therefore TRAbs can lead to GO development. The latter is characterized by profound orbital tissue remodeling, with inflammation and extracellular matrix deposition being the drivers of the clinical GO manifestations, including periorbital edema, exophthalmos, limited ocular movement, and in severe cases, optic nerve compression and blindness. Mechanistically, GO is induced by both autoantibodies and immune cell influx within the retro-orbital tissue. Orbital pre-adipocytes and fibroblasts express the TSH receptor, which, once activated by activating autoantibodies, creates a cross-talk with the insulin-like growth factor 1 receptor (IGF1R), leading to the induction of adipogenesis and differentiation of fibroblasts into myofibroblasts producing extracellular matrix (5). The net result is an expansion of the retro-orbital tissue causing eye protrusion and limited movement. In addition, orbital fibroblasts can engage with autoreactive T cells through CD40 expression, resulting in the production of cytokines and more immune cell influx (3, 4).

Both GO and GD are driven by a combination of genetic susceptibility (accounting for 79%) and environmental exposures (accounting for 21%) (6, 7). However, in recent years, gut microbiome composition and functionality have been implicated as another driver of host’s health and disease, including various autoimmune diseases (8, 9). Interestingly, the gut microbiome is predominantly influenced by environmental factors, with only 2-8% of the variation explained by the host’s genetics (10, 11).

In addition to the well-recognized cross-talk between gut commensals and the host immune system (12), there seems to be an essential bidirectional signaling axis between the gut microbiome and the local thyroid gland, regulating thyroid homeostasis by iodine uptake, degradation, and enterohepatic circulation (13). It has been shown that perturbations of the gut microbiome composition are present in human GD and GO fecal samples (14–18) and could therefore be pivotal in the pathophysiology of the disease.

Deviations in the gut microbiome have often been associated with impaired intestinal integrity and increased intestinal permeability (19, 20). This phenomenon, in popular terminology called a “leaky” gut, results in the translocation of bacterial components into the circulation, such as lipopolysaccharide (LPS), an endotoxin located in the outer membrane of many Gram-negative bacteria (21–23). The leakage of bacterial compounds may promote systemic inflammation as they are recognized by innate receptors ubiquitously expressed by immune and parenchymal cells (20). LPS-binding protein (LBP), a soluble glycoprotein that enhances the host’s immune response to endotoxins, is used as a serum biomarker of intestinal permeability (23). LBP has been previously reported to be increased in the circulation of GD patients (24).

In this observational study, we hypothesize that enhanced intestinal permeability, as inferred by measuring circulating levels of LBP, zonulin, TLR5 and TLR9 ligands, may aggravate orbital inflammation and, thus, increase myofibroblast differentiation and the degree of fibrosis. For this, we use two distinct cohorts of GO patients, one of which is a unique cohort with available fecal and blood samples as well as retroorbital adipose tissue biopsies.

## Methods

### Study population

The cross-sectional data were obtained during study visits between 2013 and 2017. All participants provided written informed consent. The study was approved by the medical ethical review board of the Amsterdam University Medical Center (Amsterdam UMC), location AMC, and followed the principles of the Declaration of Helsinki (revisions 6 and 7).

**Figure 1:**
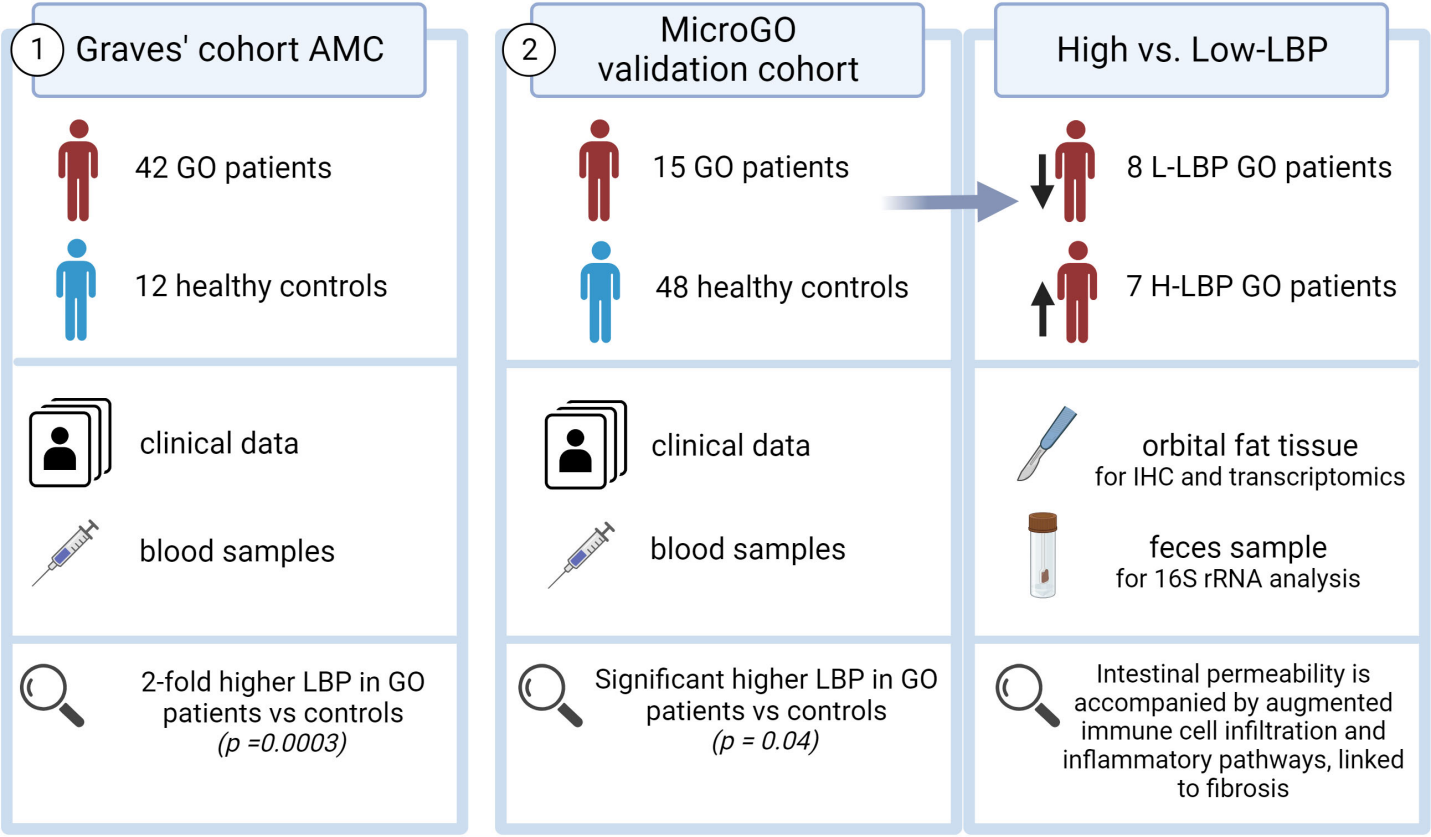
Graphical abstract.

Two distinct cohorts were used in this study (**Figure 1**). The first cohort is the “*Graves’ cohort AMC”* and comprises a cross-sectional cohort including 42 Graves’ patients with GO (N=21 with inactive moderate-to-severe GO and N=21 with active moderate-to-severe disease) and 12 healthy controls, of which clinical data and blood samples were collected. All Graves’ patients in this cohort were currently using antithyroid drugs and levothyroxine supplementation therapy (**Table 1**).

**Table 1:** Patient characteristics of MicroGO cohort subjects.

Characteristic	GO patients (N = 15)	Healthy controls (N = 48)	p-value
**Male sex (%)**	5 (33.3)	22 (45.8)	0.579
**Age (yr)**	46.47 ± 11.90	41.96 ± 12.72	0.229
**Current smoker (%)**	5 (33.3)	0 (0.0)	**<0.001**
**BMI (kg/m^2^)**	25.43 ± 4.17	23.60 ± 2.79	0.055
**TSH (mU/L)**	1.25 [0.46 – 3.57]	1.60 [1.08 – 2.60]	0.496
**FT4 (pmol/L)**	18.74 ± 3.77	15.85 ± 2.13	**0.001**
**FT3 (pmol/L)**	4.28 ± 0.59	4.96 ± 0.66	**0.001**
**TBII (U/L)**	3.42 [0.00 – 9.10]	0.00 [0.00 – 0.00]	**<0.001**
**LBP (μg/mL)**	11.13 [9.89 – 13.95]	9.06 [6.87 – 11.39]	**0.044**

For normally distributed parameters, data are presented as mean ± SD, and p values were calculated using a Student’s t-test. For non-normally distributed parameters, data are presented as median [IQR], and the p-value was calculated using the Mann-Whitney U test. Nominal variables are presented as n (%). BMI, Body Mass Index; TSH, thyroid-stimulating hormone; FT4, free thyroxine; FT3, Free triiodothyronine; TBII, thyroid-binding inhibitory immunoglobulins; LBP, LPS-binding protein.

Bold values highlights significant p-values.

The second cohort is our validation cohort, called the “*Graves’ orbitopathy (MicroGO) cohort”*. This cohort comprises a smaller group of 15 GO patients and 48 healthy controls. It includes clinical data and blood samples of both groups, and fecal specimens and biopsies of orbital fat tissues of the Graves’ patients.

All MicroGO cohort patients underwent orbital decompression surgery based on established clinical protocols from the Graves Orbitopathy outpatient clinic and were on stable antithyroid treatment for over three months (**Table 2**). During this procedure, orbital connective tissue was removed to reduce the degree of proptosis. One part of the excised orbital tissue was snap-frozen in liquid nitrogen and stored at -80°C until analysis; the other was held in 4% formalin for histological analysis. One day before the surgery, anthropometric characteristics, fasting blood samples, and stool samples were collected. Serum samples of healthy controls were collected similarly via the Amsterdam UMC Liquid Biopsy Centre and were matched to the GO patients based on gender, age, and BMI. All healthy controls were non-smokers and did not use any medication. Unfortunately, fecal samples and orbital tissue of healthy controls were not available. Individuals (GO patients and healthy controls) who underwent treatments with not eligible antibiotics, prednisone, or proton pump inhibitors within three months prior to the scheduled operation date, were not included to avoid confounding effects on the gut microbiota composition. A total of 15 GO patients and 48 healthy controls between 18-70 years old were included in the study.

**Table 2:** Patient characteristics of the MicroGO patients, separated by LBP serum level.

Characteristic	Low LBP group (N = 8)	High LBP group (N = 7)	p-value
**Male sex (%)**	2 (25.0)	3 (42.9)	0.855
**Age (yr)**	42.12 ± 12.76	51.43 ± 9.31	0.136
**Current smoker (%)**	2 (25.0)	3 (42.9)	0.855
**BMI (kg/m^2^)**	24.20 ± 2.77	26.83 ± 5.23	0.237
**TSH (mU/L)**	0.72 [0.26 - 3.54]	1.60 [1.25 - 3.35]	0.482
**FT4 (pmol/L)**	20.59 ± 3.65	16.63 ± 2.79	**0.037**
**FT3 (pmol/L)**	4.39 ± 0.35	4.16 ± 0.80	0.471
**TBII (U/L)**	1.71 [0.00 - 6.12]	5.17 [1.68 - 13.41]	0.473
**LBP (μg/mL)**	9.89 [8.15, 10.40]	14.33 [13.18, 20.81]	**0.001**
**CAS score**	3.0 [1.5 - 4.0]	2.0 [0.75 – 2.25]	0.195
**Hertel OS (mm)**	25.0 ± 2.8	22.9 ± 1.6	0.089
**Hertel OD (mm)**	21.2 ± 2.9	23.4 ± 2.3	0.211
**Thyroid medication (%)** **- Block-and-replace therapy** **- Levothyroxine only** **- No (thyroid) medication**	8 (100.0%) 0 (0.0%) 0 (0.0%)	4 (57.1) 1 (14.3) 2 (28.6)	0.117
**Severity** **- Mild** **- Moderate to severe** **- Sight-threatening**	0 (0.0%) 8 (100%) 0 (0.0%)	0 (0.0%) 8 (100%) 0 (0.0%)	1.000

For normally distributed parameters, data are presented as mean ± SD, and p values were calculated using a Student’s t-test. For non-normally distributed parameters, data are presented as median [IQR], and the p-value was calculated using the Mann-Whitney U test. Nominal variables are presented as n (%). BMI, Body Mass Index; TSH, thyroid-stimulating hormone; FT4, free thyroxine; FT3, Free triiodothyronine; TBII, thyroid-binding inhibitory immunoglobulins; LBP, LPS-binding protein; CAS, clinical activity score; OD, Oculus Dexter; OS, Oculus Sinister.

Bold values highlights significant p-values.

The 7-point Clinical Activity Score (CAS) (25) for assessing disease activity (**Table S1**) and EUGOGO classification (mild, moderate-to-severe, or sight-threatening) (**Table S2**) (26) for assessing disease severity were determined during an outpatient visit before the surgery by either an endocrinologist or an ophthalmologist.

### Thyroid markers

Serum levels of thyroid-stimulating hormone (TSH) and free thyroxine (fT4) were determined by electrochemiluminescence assay (ECLIA) using the *Cobas C8000* analyzer (Roche Diagnostics, Basel, Switzerland). Free triiodothyronine (fT3) was determined by Chemiluminescent Microparticle Immunoassay (CMIA) using the *Alinity I system* (Abbott Laboratories, Lake Bluff, Illinois, USA). Autoimmune hyperthyroidism was diagnosed by measuring serum thyroid-binding inhibitory immunoglobulins (TBII), which exploit the antibodies’ ability to inhibit labelled-TSH binding to the TSHR (27). TBII levels were determined on TRACE technology with a *Kryptor Compact Plus* analyzer (BRAHMS Thermo Scientific, Henningsdorf, Germany). Reference values ranged from 0.5-5.0 mU/L for TSH, from 12-22 pmol/L for fT4; from 2.5-5.1 pmol/L for fT3; TBII serum levels ≤1.0 U/L were considered negative, whereas TBII serum levels ≥ 1.8 U/L were considered as positive.

### Intestinal permeability markers

Concentrations of LBP in serum (in ug/ml) were assessed by ELISA (Human LBP ELISA, Hycult Biotech, Leiden, The Netherlands) accordingly to the manufacturer’s instructions. Participants were divided into either high- (H-LBP) or low-LBP (L-LBP) serum levels based on the median value of serum LBP. Zonulin serum concentrations (in ng/ml) were measured by ELISA (Human Haptoglobin DuoSet ELISA, R&D systems, Minnesota, USA) following the manufacturer’s instructions. Presence of active ligands for TLR5 and TLR9 was tested using the HEK-Blue human TLR5/TLR9 reporter cell lines (InvivoGen). For this, 20 uL serum samples were used per well (in a 96-well plate) and mixed with 180 uL of 1.4x1E5 cells/mL in HEK-Blue SEAP (secreted embryonic alkaline phosphatase) detection media (InvivoGen, San Diego, USA), which allowed the detection of SEAP after 5-hour exposure as the reporter protein is secreted by HEK-Blue cells upon TLR5/TLR9 signaling activation.

### Immunohistochemistry

Formalin-fixed paraffin-embedded (FFPE) sections of orbital biopsies were utilized for immunohistochemical staining. Slides were deparaffinized in 100% xylene and rehydrated in ethanol (100%, 96%, and 70%) and H2O, followed by blocking endogenous peroxidase in 3% H2O2 methanol for 20 minutes and heat-induced epitope retrieval (HIER) in citrate buffer pH 6.0 at 98°C for 10 minutes. FFPE sections were then incubated with primary antibodies anti-CD68 (KP1; Cell Signaling Technology, Danvers, USA), anti-CD3 (D7A6E; Cell Signaling Technology, Danvers, USA), anti-smooth muscle actin alpha (1A4 clone, DAKO), following by incubation with the secondary Poly-HRP-conjugated antibodies (BrightVision, Gothenburg, Sweden) for 30 minutes at room temperature. Staining was visualized with a 3,3’Diaminobenzidine (DAB) kit (Sigma Aldrich, St Louis, USA).

#### Image quantification

After immunohistochemistry, images of distinct areas of orbital tissue sections were taken in a blinded–manner. Staining for CD68, α-SMA (ASMA), and CD3 were quantified with the Image J software and are presented as a percentage of positive areas.

### GeoMx digital spatial transcriptome profiling

Orbital FFPE sections (8μm thick) were used to determine the transcriptional profile of specific fibrotic areas within the retro-orbital biopsies from GO patients. This assay is developed by Nanostring (NanoString Technologies Inc, Seattle, Washington, USA) and employs oligo-labeled probes (complementary sequences) that specifically align to targeted mRNA transcripts. Here we used the NanoString immune-oncology RNA targets panel, including six negative probes and five probes targeting housekeeping RNA transcripts. To compare the gene expression across multiple samples, the raw gene expression data were first normalized to the signal from negative probes and afterward to the housekeeping genes.

Several regions of interest per orbital section were selected based on immunofluorescence staining of morphology markers: DNA (nuclear staining), CD45, ASMA, and FABP4. This allowed us to navigate the slide and determine which areas were active fibrotic areas and which were occupied by mature adipocytes. As fibroblast activation and fibrosis are at the core of GO pathogenesis, we selected the region of interest within the ASMA-positive area. These selected regions were used by the GeoMx instrument for gene expression quantification.

### Gut microbiota analyses

DNA was extracted according to the in-house 16S rRNA gene-based PCR amplification protocols performed at the University of Helsinki, using primers detecting the V3 regions of the 16S rRNA genes (28). Samples were sequenced by Illumina HiSeq (Illumina, San Diego, USA). Sequences were truncated to 150 nucleotides. This read length gives accurate quality scores, which start dropping after 160-180 nucleotides, as reported previously (28). Only forward reads were processed as these are most reliable and provided an accurate prediction of taxa when tested with the mock community (see Korpela et al. (28) and the MARE package manual in R, version 1.0, https://github.com/katrikorpela/mare). The minimum read abundance (sequences that occur less frequently than the threshold were discarded to avoid sequencing errors) was set to 10^-05^. Consequently, sequences that appear fewer than 6 times were removed from preprocessing. The low threshold is set this way because of the exploratory nature of the sample type. Both databases “silva_v3v4_Gut.udb (confidence level 0)” and “silva_v3v4.fasta” were used for the annotation of OTUs. Three different non-template controls were preprocessed with the samples.

### Data availability

16S RNA gene sequencing data are deposited in the European Nucleotide Archive. The gene expression data obtained with Nanostring DSP GeoMx technology in formalin-fixed paraffin-embedded orbital tissues will be available upon request.

### Statistical analysis

Mann-Whitney U or unpaired Student’s t-tests were used to analyze differences between the two groups. Multiple comparisons (e.g., gene expression data analysis) were done by one-way ANOVA tests, followed by the Kruskal-Wallis test. Spearman nonparametric rank correlation was used to study relationships between variables. Statistically significant differences are shown by * for p-values equal or below 0,05, ** for p-values equal or below 0,01, *** for p-values equals or below 0,001.

## Results

### GO is accompanied by increased intestinal permeability

**Figure 2:**
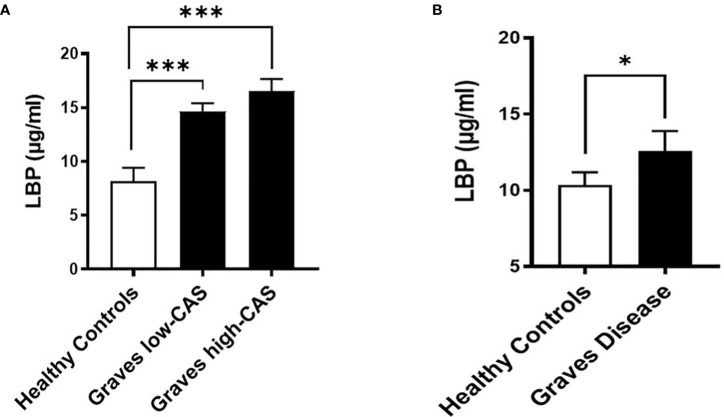
Serum LBP levels (µg/ml) in two distinct cohorts. **(A)** Graves’ cohort AMC: Patients with inactive (CAS<3) moderate-to-severe (N=21) and active (CAS ≥4) moderate-to-severe (N=21) Graves’ Orbitopathy (GO) compared to healthy controls (N=12). p=0,0003 between healthy and inactive GO, p=0,0001 between healthy and active GO groups. **(B)** Graves’ orbitopathy (MicroGO) cohort (validation cohort): GO patients (N=15) compared to healthy matched controls (N=48); p=0.04. **(A, B)**. Data shown as mean +/- SEM (standard error of the mean). Statistical significance determined with Mann-Whitney U test. * p ≤ 0.05, *** p ≤ 0.001.

To investigate whether GO is accompanied by increased intestinal permeability, we measured the LBP concentrations, a common marker of intestinal permeability, in serum samples from the *Graves’ cohort AMC*. We found that serum LBP levels are significantly increased (approximately 2-fold) in GO patients with inactive moderate-to-severe GO (N=21) and active moderate-to-severe (N=21) GO compared to healthy controls (N=12) (**Figure 2A**; **Table S3**). However, the lack of a significant difference between the two patient groups may indicate that intestinal permeability is an early event that occurs equally at the beginning of GO pathogenesis and throughout its clinical manifestations.

To determine whether increased intestinal permeability influences the inflammatory milieu in the orbital tissue, we used a different unique cohort of 15 GO patients (*MicroGO* cohort) in which biopsies of orbital tissues were taken during surgery. Measurement of LBP in serum samples from this cohort validated the increased LBP levels in amount and significance found in GO patients as compared to healthy matched controls (**Figure 2B**).

The baseline characteristics of the MicroGO cohort study (validation cohort) are provided in **Table 1**. Briefly, the majority of all 63 participants were female (57.1%), with an average age of 43.0 years. On average, the 15 GO patients were 46.5 years of age; 66.6% were female. GO patients had significantly different thyroid serum levels compared to healthy controls, with higher FT4 serum levels and lower FT3 and TSH serum levels. As expected, GO patients had significantly higher serum levels of TBII and LBP.

**Figure 3:**
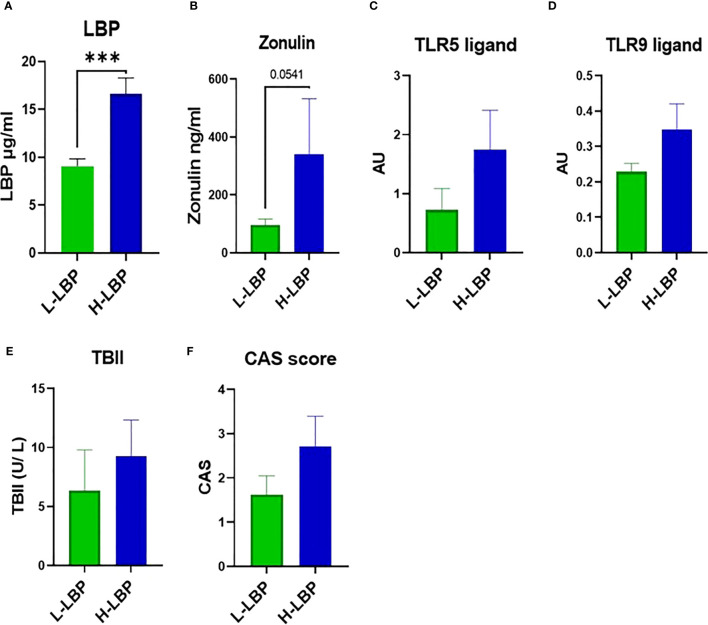
GO patients of the MicroGO cohort were divided into low- (L-LBP) versus high- (H-LBP) lipopolysaccharide-binding protein (LBP) serum levels. **(A)** Serum LBP levels (µg/ml); p=0,0003 **(B)** serum zonulin concentrations in ng/ml; p=0,054 C,D. HEK-Blues reporter activity as a proxy of circulating levels of active bacterial ligands of **(C)** TLR5 (flagellin) and **(D)** active bacterial ligands of TLR9 (unmethylated CpG motifs of bacterial DNA); **(E)** serum levels of TSH-binding inhibitor immunoglobulin (TBII) in U/L; **(F)** Clinical Activity Score (CAS). Data is shown as mean +/- SEM (standard error of the mean). Statistical significance determined with Mann-Whitney U test. *** p ≤ 0.001.

The GO group was then divided into high- (H-LBP) versus low-LBP (L-LBP) patients, based on the median serum levels LBP (11.13 ug/ml) (**Table 2**), to investigate whether differences in inflammatory and fibrotic markers within the orbital tissues occur as a result of different degrees of intestinal permeability. This resulted in eight patients with low serum levels of LBP (median 9.89 ug/ml) and seven patients with high LBP serum levels (median 14.33 ug/ml)(**Figure 3A**). No significant differences were found in TBII serum levels, Hertel measurements, EUGOGO severity classification, anthropometric parameters, and medication use between the patients with low and high LBP. Interestingly, patients of the H-LBP group showed higher levels of another gut permeability marker, serum zonulin (p=0.054), than that of the L-LBP group (**Figure 3B**). In line with higher permeability and higher translocation of bacterial components from the gut lumen to the circulation, the serum samples from the H-LBP group had a greater capacity, albeit not significant (p=0.1), to induce the activation of TLR5 and TLR9 signaling when compared to patients from the L-LBP group (**Figures 3C, D**). Importantly, no significant differences in autoantibodies titers nor in clinical features were found between patients of the two groups (**Figure 3E**; **Table 2**), indicating that any differences found in inflammatory markers in the orbital tissues are not attributable to the discrepancy in the levels of TSHR autoantibodies or different GO phenotypes, but rather due to the loss of intestinal barrier integrity. Similarly, the CAS score was not significantly different between H-LBP and L-LBP groups, suggesting that “intestinal leakage” occurs both in mild and severe GO and may be an early event in GO disease (**Figure 3F**).

### Serum LBP levels are linked to specific gut commensal bacteria

**Figure 4:**
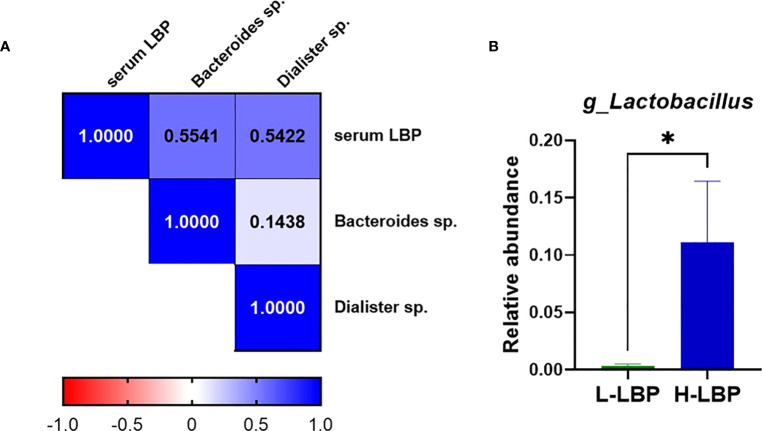
Relationship between gut microbiota and serum LBP levels in GO patients. **(A)** Heatmap showing Spearman’s correlation rho coefficients of serum LBP and the abundance of two Gram-negative species, *Bacteroides* sp.(p=0,042) and *Dialister* sp. (p=0,048) (respective annotations: Bacteroidetes_Bacteroidia_Bacteroidales_Bacteroidaceae_Bacteroides_unculturedorganismHQ761051.1.1439 and Firmicutes_Negativicutes_Selenomonadales_Veillonellaceae_Dialister_unculturedorganism); **(B)** Relative abundance of genus *Lactobacillus* (relative to total genera found in fecal microbiota) in H-LBP GO patients (N = 7) and L-LBP (N=8) p=0,033. Data displayed as mean +/- SEM (standard error of the mean). *p<0.05.

Using fecal DNA isolated from stool samples of the GO patients within our validation Graves’ orbitopathy cohort, we performed 16S rRNA amplicon sequencing to determine the taxonomic profile of the fecal microbiota. This allowed us to determine that the relative abundance of two Gram-negative species, *Bacteroides* spp. and *Dialister* spp., were positively correlated with the concentration of serum LBP (**Figure 4A**). Moreover, we found that the relative level of *Lactobacillus* spp. was very strongly (over 10-fold) increased in the H-LBP group (**Figure 4B**). Notably, *Lactobacillus* abundance in stool samples was shown to be associated with the severity of GO and specifically with orbital adipogenesis (29).

### Higher intestinal permeability is accompanied by augmented immune cell infiltration and inflammatory pathways in orbital tissues

**Figure 5:**
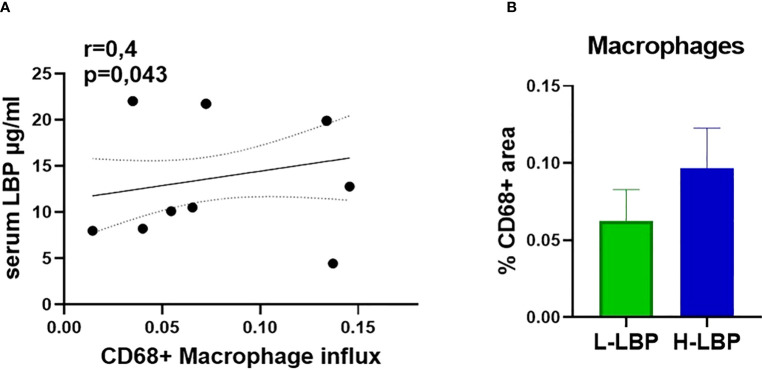
Augmented infiltration of CD68+ macrophages in GO patients is correlated to serum LBP levels. **(A)** Spearman correlation between the influx of CD68+ macrophages (shown as percentage of CD68-positive areas) in the orbital tissue and serum LBP levels of GO patients (N=15), p = 0.043; **(B)** Influx of CD68+ macrophages in L-LBP (N=8) vs H-LBP (N=7) GO patients.

Immunohistochemistry of formalin-fixed paraffin-embedded orbital sections was employed to quantify the influx of CD68-positive macrophages. We found a significant positive correlation between the influx of macrophages (CD68+) in the orbital tissue and serum LBP level (r = 0.4, p = 0.043; **Figure 5A**), linking a “leaky” gut with orbital inflammation. However, the increased influx of macrophages in the H-LBP group was not statistically significant (**Figure 5B**).

In line, the influx of macrophages was also significantly correlated with active myofibroblasts, assessed by immunostaining for α-smooth muscle actin (ASMA) (r = 0.5, p = 0.011), whereas ASMA was significantly correlated (r = 0.4, p = 0.043) with CD3 T cells as well (**Figures 5A, B**).

**Figure 6:**
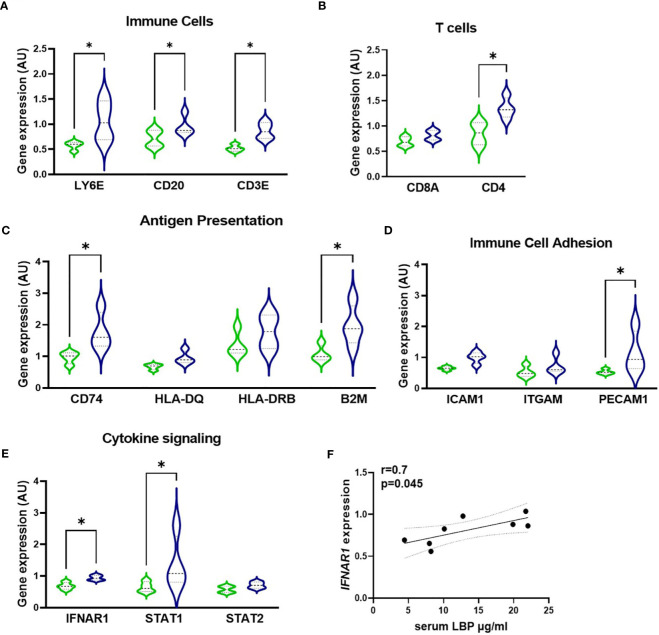
Gene expression profile within the active fibrotic ASMA-positive area of orbital tissue of L-LBP (green, N=8) versus H-LBP (blue, N=7) GO patients. **(A)** Gene expression of immune cells markers LY6E (neutrophil granulocytes), CD20 (B lymphocytes), and CD3E (T lymphocytes); **(B)** Gene expression of T-cells markers CD8a and CD4; **(C)** Gene expression of antigen presentation cells markers CD74, HLA-DQ, HLA-DRB, and B2M; **(D)** Gene expression of Immune Cell Adhesion and Migration markers ICAM1, ITGAM, and PECAM1 as markers; **(E)** Gene expression of cytokine signaling markers IFNAR1, STAT1, and STAT2; A-E. Data shown as mean =/- SEM; gene expression assessed by GeoMx digital spatial profiler technology, raw data normalized for negative probes and housekeeping gene; p<0,05. **(F)** Significant Spearman correlation between serum LBP levels and IFNAR1 gene expression in GO patients (N = 15), p = 0.045, r=0.7. Statistical significance determined by one-way ANOVA tests, followed by the Kruskal-Wallis test. *p<0.05.

Next, to quantify the relationship of serum LBP levels with the inflammatory profile of GO patients, the GeoMx Digital Spatial profiler technology was used to profile the gene expression specifically within the active fibrotic area (ASMA-positive) of the orbital tissue, which was enriched with CD45-positive immune cells. This enabled the simultaneous quantitation of multiple inflammatory genes in a specific area of interest. (**Figures 6**, **S1**). Similarly to the macrophage influx, expression of immune cell markers *LY6E*, *CD20*, *CD3*, *CD4*, and *CD8* showed that increased intestinal permeability is accompanied by enhanced recruitment in the orbital tissue of granulocytes, B cells, and T cells (specifically CD4+ T cells) as the H-LBP group displays a marked increase in the expression of these phenotypic immune cell markers (**Figures 6A, B**). In line, the expression of genes involved in antigen presentation (both via MHC class I and II, *B2M* and *CD74*, respectively) and immune cell adhesion (*PECAM1*) were significantly upregulated in patients of the H-LBP group compared to those of the L-LBP group (**Figures 6C, D**). In addition, patients of the H-LBP group displayed an enhanced expression of genes involved in the type 1 interferon pathway, such as interferon-alpha/beta receptor and signaling molecule STAT1, and the expression rate of interferon-alpha and -beta receptor subunit 1 (IFNAR1) positively correlates with the serum concentration of LBP (**Figures 6E, F**). Notably, this pathway is important in autoimmunity as it boosts antigen presentation.

### Orbital inflammation is linked to fibroblast activation

**Figure 7:**
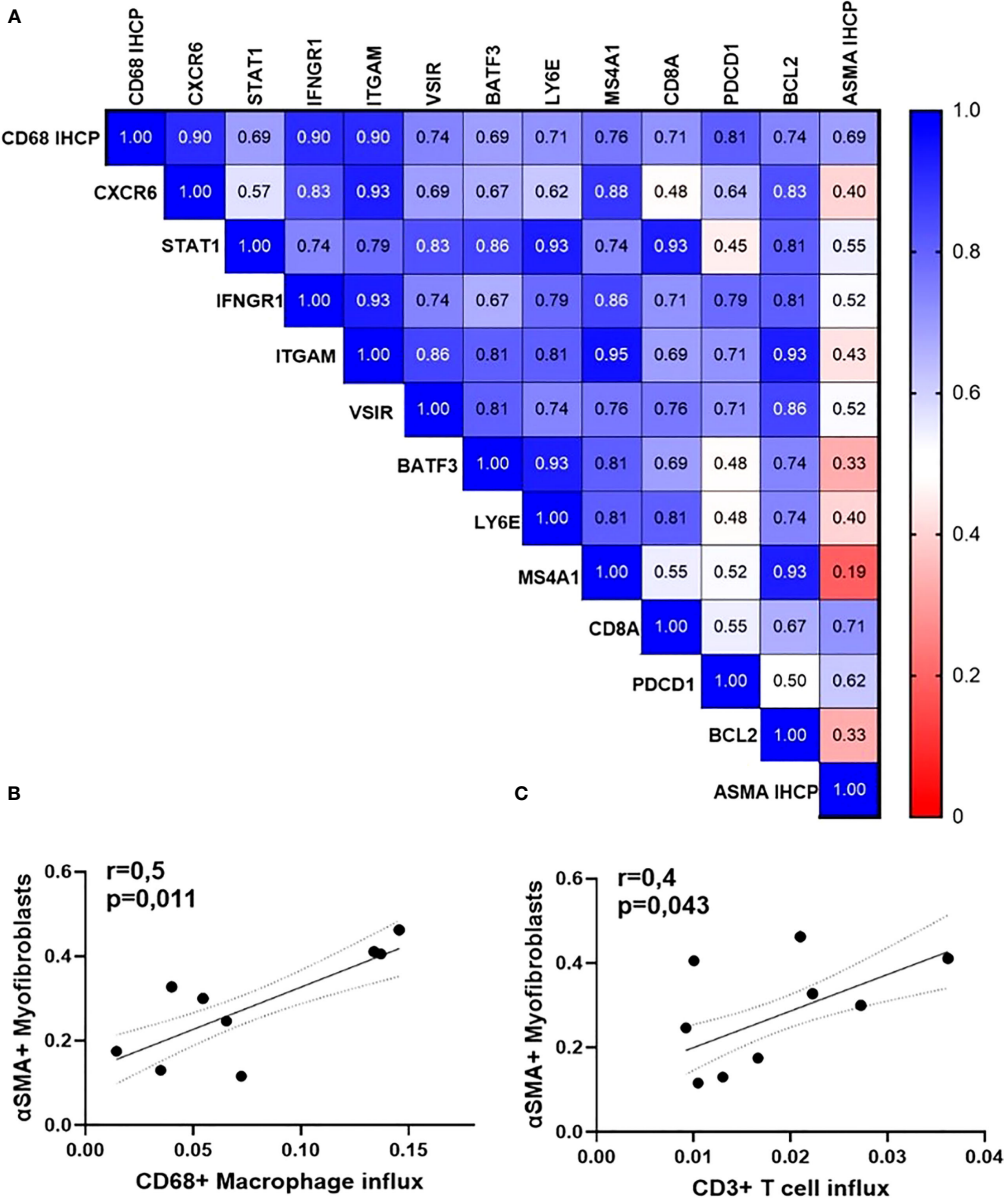
Orbital inflammation is linked to fibroblast activation in GO patients (N=15). **(A)** Heatmap of Spearman’s rho rank correlation coefficients between gene expression rates, influx of macrophages, and accumulation of myofibroblasts; **(B)** Spearman correlation between the number of orbital macrophages and CD3 T lymphocytes; **(C)** Spearman correlation between CD68+ macrophages (p=0,011, r=0,6) and ASMA-positive myofibroblasts (p=0,043, r=0,4).

Lastly, we found that the rate of macrophage influx (assessed by immunohistochemistry for CD68) is positively associated with higher expression of genes encoding for protein pivotal in cytokine signaling, cell adhesion, apoptosis, as well as with the percentage of active α-smooth muscle actin-positive myofibroblasts (detected by immunohistochemistry) (**Figure 7A**). The latter are active secretors of collagen and contribute to the expansion of the retro-orbital tissue. The number of myofibroblasts within the orbital tissue was significantly and positively associated with the influx of macrophages (r = 0.5, p = 0.011) and T cells (**Figures 7B, C**).

As persistent inflammation is known to be linked to fibrosis development (30), here we reveal that increased gut permeability is associated with the immune cell infiltration of orbital tissue in GO, the degree of local inflammation, and the differentiation of fibroblasts in ASMA-positive myofibroblasts, which actively secrete extracellular matrix.

## Discussion

In this pilot study, we have demonstrated a positive link between increased intestinal permeability and local inflammation and fibrosis within the orbital tissue of GO patients.

The occurrence of a “leaky gut” during Graves’ orbitopathy is supported by a previous study that found significantly elevated serum levels of LPS, I-FABP, zonulin, and D-lactate in patients with initial GD compared to healthy controls (24).

The zonulin family peptide is a potent regulator of intercellular tight junctions of the intestinal epithelium. Mucosal defects can lead to increased serum zonulin levels and can be used as a leaky intestinal barrier, dysbiosis, and inflammation biomarker (31, 32). GO patients with high serum LBP levels also had elevated zonulin levels. In line with this finding, bacterial ligands of Toll-like receptors 5 and 9, namely flagellin and unmethylated CpG motifs of bacterial DNA, appeared to be increased in the circulation of obese patients supporting the hypothesis that intestinal barrier dysfunction leads to the translocation of bacterial components into the bloodstream (33).

The thyroid biomarkers TSH, FT4, and FT3 were all within the normal reference range in these patients, indicating that the intestinal leakage persists after achieving a euthyroid state. We did not compare our subjects based on the serum levels of thyroid biomarkers or CAS score since these are values to identify the GO phase, while orbitopathy may remain despite remission of Graves’ disease, revealed by the TBII-negative serum levels in some patients.

Immunohistochemistry and Multiplexed RNA *in-situ* hybridization via NanoString digital spatial profiling technology enabled the assessment of the degree of immune cell recruitment and inflammatory processes activated in GO tissues. Moreover, we could link orbital inflammatory markers with the rate of intestinal permeability as well as the degree of myofibroblast expansion, which causes the clinical GO manifestations. In GO, fibroblasts are activated by TSHR-binding autoantibodies and can differentiate into adipocytes or myofibroblasts, with consequent extracellular matrix deposition and retro-orbital tissue expansion (3). For this reason, when investigating the degree of inflammation and the differences in gene expression between patients in the low- and high-LPB groups, we selected the ASMA-positive regions, which are active fibrotic regions and showed enrichment in CD45-positive leukocytes as compared to the area occupied by mature adipocytes only.

We demonstrated a positive association between serum LBP levels and macrophage influx in GO orbital tissues, establishing a link between “leaky gut” and macrophage influx. Interestingly, a recent paper demonstrated distinct macrophage immunophenotypes in GO orbital tissues, with M1-like proinflammatory macrophages being predominant in active GO and M2-like anti-inflammatory macrophages dominating in stable GO (34). The expression of IL-6 and transforming growth factor-ß was found respectively higher in M1 and M2 macrophages. However, in our study, these markers were not significantly different between high- and low-LBP groups possibly indicating a similar macrophage composition in GO tissues, although this would require future targeted investigations. In addition to macrophages, the expression of granulocyte, B cell, and T cell markers was significantly elevated with higher intestinal permeability. Particularly CD3+ T cell infiltration was found to be linked to the accumulation of myofibroblasts and postulated to be a central player in GO tissue remodeling (35). Indeed, T cells have been shown to infiltrate the retro-orbital tissue at an early stage of GO in humans (36) and analysis of antigen receptor variable region repertoires has shown that the autoreactive T cells infiltrating the thyroid gland are found in retroorbital tissues (37). We may speculate that the systemic inflammation caused by intestinal permeability aids the infiltration of TSHR-reactive T cells into secondary organs. Various studies have recently reported significant associations between the relative abundances of the gut microbiota and diagnostic parameters of thyroid status and thyroid antibodies in patients with Graves’ disease (38–45) and/or Graves’ orbitopathy (17, 46, 47) compared to healthy controls. Interestingly, similar to our results, Shi and colleagues showed an increased abundance of *Bacteroides* sp. and Lactobacillus in GO patients (17). In a recent study, *Bacteroides* spp. was identified as one of the top bacterial biomarkers for predicting the severity of GO and was significantly correlated to TSH and FT4 levels, however the whole Bacteroides phylum was decreased in the GD group (18). Given the observational study design, conclusive evidence of a causal relationship or its direction cannot be drawn from our study. Specifically, it remains unclear whether the bacterial species *Bacteroides* spp. and *Dialister* spp., identified to correlate with LBP levels, play an active role in triggering gut barrier permeability.

Two recent studies have investigated the effect of transplanting gut microbiota of GD/GO patients in a GD/GO mouse model (42, 48). The gut microbiota composition of medication-naïve GD patients differed significantly from healthy controls, which led to a higher disease incidence in mice after fecal microbiota transplantations (FMTs) from these GD patients compared to mice treated with FMT from healthy human controls (73.3% vs. 28.6%, respectively, p = 0.03) (42). A second study showed significant variation in gut microbiota composition in murine models of GD/GO correlating with GO heterogeneity, including enlarged volume of orbital brown adipose tissue (48). Analysis of fecal microbiota profiles revealed an increased Bacteroides to Firmicutes ratio in severe GO patients versus healthy controls. Assuming that the GO patients in this study also had elevated LBP levels, this would confirm our present finding that the abundance of *Bacteroides* sp. was correlated with high LBP levels. The fecal samples of the GO patients were transferred via FMT into mice immunized with human thyrotropin receptor (as a GD model), resulting in a hyperplastic thyroid and increased fat area in the middle orbital tissue. Here, an inverse correlation between the relative abundance of *Lactobacillus* spp. and TRAb was observed in the FMT-treated mice. In line, another study showed significant enrichment of *Lactobacillus* spp. in GO mice (p = 0.018), positively correlated with orbital adipogenesis and serum fT4 (29). These findings are in line with our results showing that the H-LBP patients displayed a higher relative *Lactobacillus* spp. abundance and simultaneously a higher rate of activated orbital fibroblasts. However, the mice results should be interpreted with caution as the *Lactobacillus* levels in mice are much higher than those in humans (49).

A notable strength of our study is the combination of biopsies from the orbital tissue with serum and fecal samples, which allowed us to link intestinal permeability with a distant organ. The Digital Spatial Profiling technology enabled us to quantify the inflammatory gene expression in the selected regions of active fibrosis within orbital biopsies. Of note, active fibrosis is a pivotal process in the pathogenesis of Graves’ orbitopathy, yet it cannot be included in the clinical activity score and is not associated with the CAS severity. Indeed, GO disease may appear clinically quiescent and, yet, exhibit certain features of the disease, such as those seen in EUGOGO (26). In our study, the degree of fibrosis could not be accurately compared with other clinical indicators, such as serum thyroid hormone levels, as nearly all patients were taking thyroid medication on the day of surgery (as shown in **Table 2**).

Previous studies revealed that GO phenotype features were linked to thyroid autoantibody serum levels (50, 51). Both TSAb and TBII levels were significantly associated with a higher CAS score and more severe proptosis. However, our study did not find any correlation between LBP and these clinical features of GO, suggesting that variations in inflammatory markers in the orbital tissues are not attributed to differences in levels of thyroid autoantibodies or GO phenotypes and underscoring a potential role of a “leaky” gut in orbital inflammation.

As orbital decompression surgery is a last resort in treating GO (25), it remained difficult to retrieve a large cohort. Moreover, orbital surgery is only performed after extensive initial medical treatment with anti-thyroid medications and steroids, which most likely influences the gut microbiota composition. It is yet not known whether our finding of enhanced intestinal permeability in GO patients is associated with local orbitopathy or just an accompanying sign of autoimmune hyperthyroidism. Fecal samples from GD patients without eye disease, as well as mild medication-naïve inactive GO patients, might be an interesting source to investigate whether gut microbiota are a key factor in the pathogenesis of GO/GD.

This explorative study shows a positive association between gut permeability and orbital inflammation, which in turn is linked to fibrosis. Nonetheless, the causative relationship of this phenomenon has to be further studied in further intervention trials. Indeed, a human randomized clinical trial employing fecal microbiota transplantation from healthy donors in GD patients versus placebo would help uncover whether the gut microbiota are a causative factor in the onset of GD/GO pathogenesis or whether it merely reflects the effects of the disease itself. These and other interventions could be an alternative way to show that compromised barrier function is a causative factor in GD/GO pathogenesis. Such studies not only would address the possibility that a perturbed microbiome is causing GD on its own but also may aggravate or accelerate disease progression by influencing systemic immune responses.

## Conclusion

In conclusion, this study shows that GO is associated with enhanced intestinal permeability and the degree of fibrosis positively correlates with higher inflammatory tone within the orbital tissue of GO patients. Particularly, patients with high LBP serum levels presented an increased expression of genes involved in antigen presentation, immune adhesion, IFN-α signaling, and immune cell markers of macrophages, B, and T cells. This suggests that in patients with enhanced intestinal permeability, the subsequently increased translocation of bacterial compounds to the systemic circulation triggers a local inflammatory immune response in the orbital tissue. These initial findings warrant further exploration in larger GD/GO cohorts to assess how (and why) specific inflammatory pathways (e*.g.*, type I interferon and antigen presentation) in the orbital tissue correlate with gut microbiota composition and provide a basis for developing microbiota-targeting therapeutic interventions.

## Data availability statement

The data presented in the study are deposited in the European Nucleotide Archive (ENA) repository, accession number PRJEB65449.

## Ethics statement

The studies involving human participants were reviewed and approved by Amsterdam Medical Ethics Committee. The patients/participants provided their written informed consent to participate in this study.

## Author contributions

AF and ER contributed to patient selection, data analysis, and laboratory procedures and wrote the manuscript. SH contributed to the performance of immunohistochemistry on orbital tissues. WdV provided the microbiota analysis by r16S-sequencing. AvdS contributed substantially to the content discussion and edited the manuscript before submission. ER and MN reviewed the manuscript before submission. All authors contributed to the article and approved the submitted version.

## Supplementary material

Supplementary Figure 1 Regions Of Interest (ROIs) of orbital tissue of GO patients, using Multiplexed RNA *in-situ* hybridization via NanoString digital spatial profiling technology.

## Glossary

**Table:**

ASMA	α-smooth muscle actin; active fibrotic areas
B2M	beta-2-microglobulin; MHC-I molecule
BATF3	BCL2 apoptosis regulator
CAS	clinical activity score
CD3E	T lymphocytes
CD68	Macrophages
CD74	major histocompatibility complex, class II invariant chain; antigen presentation
CD8A	cytotoxic T lymphocytes
CXCR6	C-X-C motif chemokine receptor 6; T helper lymphocytes
GD	Graves’ disease
GO	Graves’ orbitopathy
H-LBP	GO patients with high serum LBP levels
HLA-DQ/DRB	class II major histocompatibility complex; DQ alpha 1/DR beta 4
ICAM1	intercellular adhesion molecule 1
IFNAR	interferon-alpha and -beta receptor subunit 1
IFNGR1	interferon-gamma receptor 1
IHC	immunohistochemistry
ITGAM	integrin subunit alpha M = CD11b; leukocyte adhesion
L-LBP	GO patients with low serum LBP levels
LY6E	lymphocyte antigen 6 family member E, neutrophils
MS4A1	CD20; B lymphocytes
PDCD1	programmed cell death 1
PECAM1	platelet and endothelial cell adhesion molecule 1
STAT 1/2	signal transducer and activator of transcription 1/2
T3	tri-iodothyronine
T4	thyroxine
TBII	TSH-binding inhibitor immunoglobulin
TLR5	flagellin
TLR9	unmethylated CpG motifs of bacterial DNA
TRAb	autoantibodies to the thyrotropin receptor
TSAb	thyroid stimulating antibody
TSH(R)	thyroid stimulating hormone (receptor)
