# Supplementary figures and images for: Intestinal permeability is associated with aggravated inflammation and myofibroblast accumulation in Graves’ orbitopathy: the MicroGO study
Source: Front Endocrinol (Lausanne). 2023 Nov 30;14:1173481. doi: 10.3389/fendo.2023.1173481 (PMC10724020; doi:10.3389/fendo.2023.1173481)

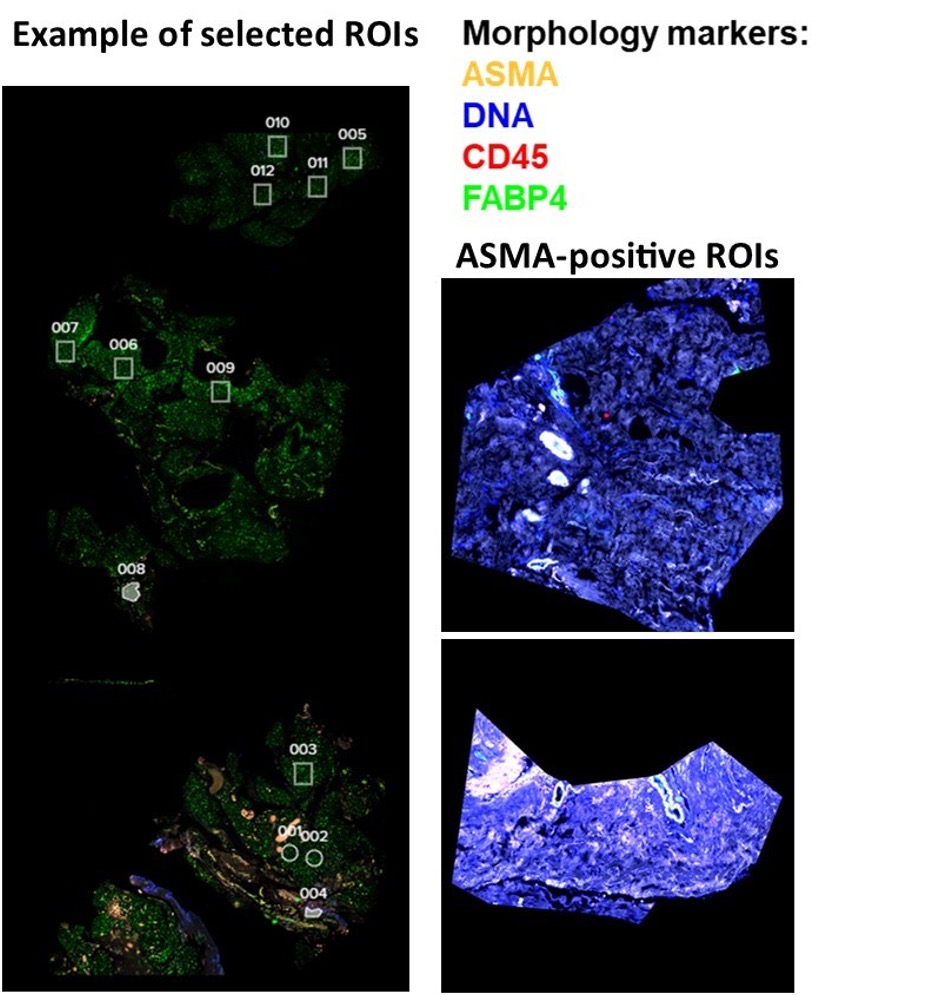

Supplement: Supplementary Figure 1 — Regions Of Interest (ROIs) of orbital tissue of GO patients, using Multiplexed RNA in-situ hybridization via NanoString digital spatial profiling technology. [file Image_1.jpeg]
