# Supplementary material for: Intestinal permeability is associated with aggravated inflammation and myofibroblast accumulation in Graves’ orbitopathy: the MicroGO study
Source: Front Endocrinol (Lausanne). 2023 Nov 30;14:1173481. doi: 10.3389/fendo.2023.1173481 (PMC10724020; doi:10.3389/fendo.2023.1173481)
Supplement: Supplementary file 3 [file Table_1.docx]

Table S1. Components of the Clinical Activity Score (CAS)

| Components of the Clinical Activity Score (CAS) |
| --- |
| Spontaneous retrobulbar pain |
| Pain with eye movement |
| Redness of the eyelids |
| Redness of the conjuctiva |
| Swelling of the eyelids |
| Swelling of the caruncle |
| Conjuctival edema (chemosis) |

The CAS score is calculated according to the presence or absence of the characteristics listed. One point is given for each presence of the characteristics listed; with 0 – 3 scored as low CAS score and 4 to 7 as a high CAS score.
