# Supplementary material for: Intestinal permeability is associated with aggravated inflammation and myofibroblast accumulation in Graves’ orbitopathy: the MicroGO study
Source: Front Endocrinol (Lausanne). 2023 Nov 30;14:1173481. doi: 10.3389/fendo.2023.1173481 (PMC10724020; doi:10.3389/fendo.2023.1173481)
Supplement: Supplementary file 4 [file Table_2.docx]

Table S2. Classification of severity of Graves' orbitopathy (GO), according to the guidelines of the 2021 European Group on Grave's orbitopathy (EUGOGO), as reported by Bartalena and colleagues^26^.

| **Classification** | **Features** |
| --- | --- |
| Mild GO | Patients whose featuer of GO have only a minor impact on daily life that have insufficient impact to justify immunomodulation or surgical treatment. They usually have one or more of the following:   - Minor lid retraction (<2mm); - Mild soft-tissue involvement; - Exophthalmos <3mm above normal for race and gender; - No or intermittent diplopia and corneal exposure responsive to lubricant. |
| Moderate-to-severe GO | Patients without sight-threathening GO whose eye disease has suffient impact on daily life to justify the risks of immunouppresion (if active) or sugical intervention (if inactive). They usually have two or more of the following:   - Lid retraction ≥2mm - Moderate or severe soft-tissue involvement - Exophthalmos ≥3mm above normal for race and gender; - Inconstant or constant diplopia. |
| Sight-threathening (very severe) GO | Patients with dysthyroid optic neuropathy and/or corneal breakdown |
