# Supplementary material for: Intestinal permeability is associated with aggravated inflammation and myofibroblast accumulation in Graves’ orbitopathy: the MicroGO study
Source: Front Endocrinol (Lausanne). 2023 Nov 30;14:1173481. doi: 10.3389/fendo.2023.1173481 (PMC10724020; doi:10.3389/fendo.2023.1173481)
Supplement: Supplementary file 5 [file Table_3.docx]

Table S3. Patient characteristics of moderate-to-severe Graves’ disease patients of the Graves’ cohort AMC, divided by the clinical activity.

| **Characteristic** | **Inactive GO (N = 21)** | **Active GO (N = 21)** | **p-value** |
| --- | --- | --- | --- |
| Age (years) | 61±11 | 61±11 | 0.97 |
| BMI (kg/m^2^) | 25.3±4.8 | 25.9±5.3 | 0.62 |
| CAS score | 0.8±0.65 | 4.9±0.87 | **<0.0001** |
| Hertel OS (mm) | 20±4 | 23±5 | **0.001** |
| Hertel OD (mm) (mm)rightright(mm) | 20±4 | 23±4 | **0.001** |
| TSH (mE/L) | 2.58±2.66 | 3.26±7.22 | 0.58 |
| FT4 (pmol/l) | 17.4±9.4 | 16.6±6.7 | 0.66 |
| T4 (nmol/l) | 116±51 | 120±35 | 0.68 |
| T3 (nmol/l) | 2.0±0.7 | 2.1±0.7 | 0.80 |
| T3 uptake | 1.06±0.16 | 1.01±0.14 | 0.12 |
| FT4 index | 124±75 | 122±43 | 0.91 |
| AntiTPO (kU/L) | 593±906 | 876±1170 | 0.23 |
| TBII (E/L) | 9.4±16.6 | 25.7±38.9 | **0.02** |
| Glucose (mmol/l) | 5.6±0.8 | 5.9±1.3 | 0.26 |
| Alkalic Phosphatase (U/L) | 92±49 | 84±28 | 0.36 |
| Gamma-GT (U/L) | 31±25 | 32±23 | 0.94 |
| LBP (µg/mL) | 15.70±5.83 | 16.26±5.20 | 0.66 |

For normally distributed parameters, data are presented as mean ± SD, and p values were calculated using a Student’s t-test. Inactive GO was considered as a CAS score < 3. Active GO was considered as a CAS score ≥4. All patients were classified as moderate-to-severe GO.
